# Radiofrequency catheter ablation for re-do procedure after single-shot pulmonary vein isolation with pulsed field ablation for paroxysmal atrial fibrillation: case report

**DOI:** 10.3389/fcvm.2024.1376229

**Published:** 2024-05-02

**Authors:** Xinyan Yang, Mingjie Lin, Yan Zhang, Juntao Wang, Jingquan Zhong

**Affiliations:** ^1^ State Key Laboratory for Innovation and Transformation of Luobing Theory, and Key Laboratory of Cardiovascular Remodeling and Function Research, Chinese Ministry of Education, Chinese National Health Commission and Chinese Academy of Medical Sciences, and Department of Cardiology, Qilu Hospital of Shandong University, Jinan, China; ^2^ Department of Cardiology, Cheeloo College of Medicine, Qilu Hospital (Qingdao), Shandong University, Qingdao, China

**Keywords:** atrial fibrillation, catheter ablation, pulsed field ablation, re-do procedure, reconnection

## Abstract

**Background:**

Catheter ablation is frequently used to manage recurrent atrial fibrillation (AF) resistant to drug therapy, with pulmonary vein isolation (PVI) as a key tactic. Pulsed field ablation (PFA) has emerged as an innovative technology for PVI but poses challenges for redo procedures.

**Case presentation:**

We report on a 73-year-old female patient who experienced recurrent AF after initial successful PVI using a novel PFA technology and subsequently underwent radiofrequency catheter ablation during a repeat intervention. The reconnection of pulmonary veins was discovered primarily in the anterior region of the right superior PV and the superior portion of the left superior PV. An anatomically-based segmental approach and larger circumferential PVI, followed by additional linear ablations at non-PV trigger sites, proved decisive in preventing further recurrence of atrial tachycardia.

**Conclusion:**

While PFA exhibits promise as a secure and efficient modality for PVI, it necessitates excellent contact quality to ensure lasting results. For patients experiencing AF recurrences post-PFI, expanded strategies incorporating both comprehensive PVI and linear ablations at targeted non-PV sites might enhance treatment outcomes.

## 1 Introduction

Catheter ablation is often recommended as the primary treatment for patients with symptomatic, recurrent atrial fibrillation (AF) that has not responded to drug therapy (1). Achieving effective and lasting pulmonary vein isolation (PVI) remains the cornerstone of AF ablation (2, 3).

Pulsed field ablation (PFA) represents an innovative technology designed for PVI, offering the distinct advantage of selective electroporation (4).

Despite these advances, there is currently no established consensus or strategy concerning redo procedures following initial single-shot PVI using PFA. In this report, we discuss a case involving a patient with recurrent AF who underwent radiofrequency catheter ablation after initially receiving PVI with PFA.

## 2 Case report

A 73-year-old female patient, presenting with recurrent episodes of palpitations, was diagnosed with paroxysmal AF one month prior to her admission to the hospital. She has no history of other cardiovascular conditions. She underwent an initial catheter ablation procedure using PFA technology. PVI was achieved with a lasso-shaped PFA catheter (Shineyo Medical). The position and attachment of the catheter are determined based on pulmonary venography. The generator outputs ranged between 1,000 and 1,500 V per application. Each vein was treated with (5) applications following the instructions of catheter. Verification of entrance and exit blockages was carried out using the PFA catheter in its expanded form at the ostia of the pulmonary veins. Repeated atrial electrical stimulation cannot induce rapid atrial tachycardia. Post-procedurally, the patient was prescribed a daily regimen of rivaroxaban at a dose of 15 mg and amiodarone at a dose of 200 mg.

**Figure 1:**
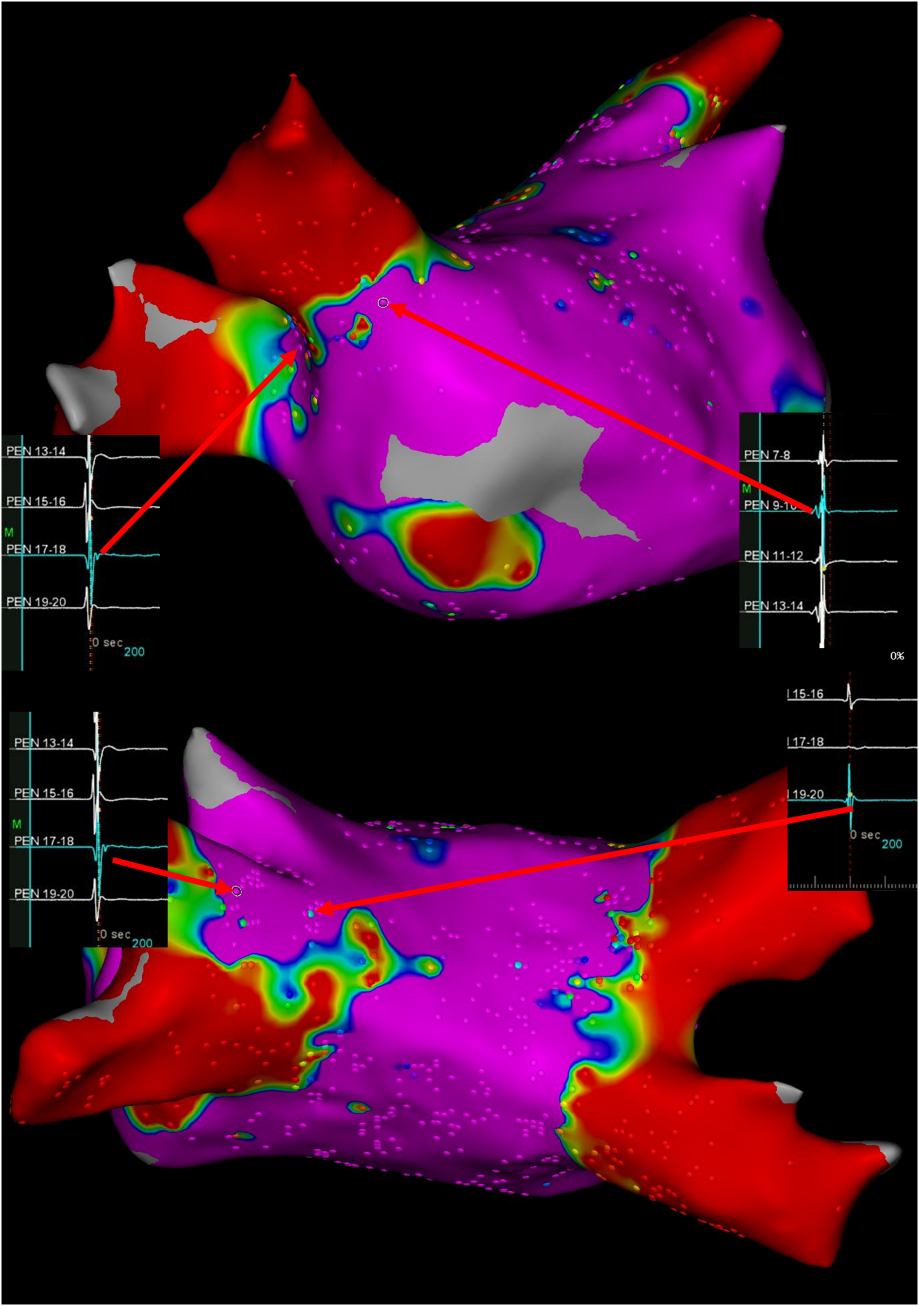
Ultra-high-density bipolar voltage maps and visualization of gaps at time of re-do procedure. Arrows depict the potential of pulmonary vein antrum. Color coding magenta is bipolar voltage >0.5 mV and red is bipolar voltage <0.1 mV.

During the outpatient appointment ten months post-ablation, the 12-lead electrocardiogram displayed a recurrence of atrial fibrillation with a ventricular rate at 145 beats per minute. Re-do procedure was performed under minimal sedation and local anesthesia. 3D electroanatomic mapping system was used for re-do procedure (CARTO3 V6, Biosense Webster). A multipolar mapping catheter (PentaRay, Biosense Webster) was used to evaluate electrical conduction during sinus rhythm. The reconnections of PVs were mainly distributed in the anterior region of the right superior PV, the superior portion of the left superior PV. Special PV potentials were found at the edge of initial ablation line (Figure 1). To achieve complete and durable PVI, an anatomically-based, potential-guided segmental approach and larger circumferential PVI was performed by a standard irrigated ablation catheter (ThermoCool SmartTouch SF catheter, 43°C, 45W, NAVI-STAR; 4-mm-tip; Biosense Webster). After completing PVI, AF was still inducible by atrial burst pacing. Therefore, we performed linear ablations of the posterior wall, i.e., roof and bottom empirically. During the linear ablation, the tachycardia was terminated. Considering that the patient was undergoing a second procedure, the superior vena cava is a common site of focal triggers, so we also performed an ablation. Finally, the atrial tachycardia could not induce with atrial burst pacing. Six months following the repeat procedure, the patient remained free of any recurrence of atrial tachycardia.

## 3 Discussion

Durable PVI remains the goal of AF ablation strategies. Rates of PVs reconnections following PVI employing either cryoballoon or radiofrequency ablation have been reported to vary widely, ranging from 22 to 38 percent and reaching as high as 62.5 percent in some cases (6).

PFA is an emerging ablation technology that, while promising, still experiences varying degrees of PV reconnection. Studies by Federico et al. and the IMPULSE trial revealed disparate rates of durable isolation using PFA, with 64.2% and 96% respectively (7–9). In the latest study by Rocca et al, the 1-year freedom from any atrial tachyarrhythmia was comparable among patients with PFA, cryoballoon, and radiofrequency ablation (10). Significant lower PV reconnection rate was observed after PFA (19.1%) during repeat ablation. These studies indicate that PFA can achieve more sustained pulmonary vein isolation rates; however, further exploration of ablation strategies is needed to improve the success rate of PFA ablation.

Federico et al. have demonstrated the prevalent sites of PV reconnections in patients undergoing repeat procedures, as identified by 3D electroanatomic mapping. In their study, 19 out of 53 patients (35.8%) exhibited such reconnections (9). The most frequently observed areas for PV reconnection were found to be the anterior region of the right superior PV, the posterior-inferior segment of the right inferior PV, and the posterior-superior portion of the left superior PV. These findings align with those noted in our case (6, 9). The geometry of the PFA catheter and the applied contact force may also play crucial roles during ablation procedures with PFA (8). The employment of intracardiac echocardiography potentially enhances the safety and effectiveness of ablation procedures. However, reported by Russo et al, PFA can be executed with consistent speed, security, and efficacy. Employing ICE for guidance during PVI did not demonstrate an enhancement in procedural metrics (11).

Re-do strategies after initial single-shot PVI with PFA are still being refined. At present, the approaches to re-do procedures typically involve additional ablation using one of several modalities: cryoballoon, radiofrequency, or a repeat PFA. When it comes to repeat ablations involving either the cryoballoon or PFA methods, challenges may arise due to catheter designs possibly causing inadequate tissue contact in previously treated areas during subsequent interventions. On the other hand, radiofrequency catheter ablation has the potential to be advantageous as a more targeted approach for re-do procedures.

Cardiac electrophysiology experts have assessed various approaches for subsequent interventions following initial cryoballoon and radiofrequency ablation treatments using radiofrequency catheter ablation. These strategies encompass either solely re-isolating the PV in a segmental fashion or implementing an anatomically-driven PVI alongside targeted, segmental isolation based on electrical potential signals. This tailored approach has been deemed to be both safe and efficacious (12).

The superior vena cava, posterior wall, coronary sinus, vein of Marshall, and the annuli of both the mitral and tricuspid valves are frequently identified as non-pulmonary vein (non-PV) trigger sites. Non-PV triggers tend to be more prevalent in certain populations, such as females, older individuals, patients with obstructive sleep apnea, and those with hypertrophic cardiomyopathy. During repeat ablation procedures, it is crucial to target these areas when a trigger has been pinpointed. Empirical ablation may also be justifiable in particular scenarios to enhance the likelihood of maintaining a rhythm free of atrial arrhythmias (13). Based on our case analysis, we posited that re-isolating PVs—with an expanded approach tailored anatomically and directed by mapping potentials—could prove beneficial. This strategy would include segmental approaches and linear ablations such as isolating the posterior wall, creating lines at the mitral isthmus and cavotricuspid isthmus, and isolating the superior vena cava, all of which should be based on potential map guidance.

## 4 Conclusions

While PFA is recognized as a secure and efficient technology, it requires optimal contact to boost the rate of effective isolation. In cases where patients experience recurrent AF following PFA, adopting an expanded PVI strategy paired with supplemental linear ablation may prove to be an effective tactic. However, additional research is required to assess the histological alterations that follow radiofrequency ablation during repeat interventions post-PFA.

## Data Availability

The original contributions presented in the study are included in the article/Supplementary Material, further inquiries can be directed to the corresponding authors.
